# The Notification Act

**Published:** 1897-12-11

**Authors:** 


### THE NOTIFICATION ACT.

Some of the proposals which are being put forward for the amendment of the Notification of Infectious Diseases Act should be looked on with considerable suspicion. They are the work of sanitary authorities, and partake of the bias natural to their origin. There can be no doubt as to the great advantage which the public derives from the notification of infectious diseases, and in view of the fact that the Act has already been put in force over about 27,000,000 out of the total 29,000,000 inhabitants of England and Wales, it seems reasonable enough to urge that it should be made compulsory all over. When, however, it is proposed to whittle away the small fees which are now paid for the notifications, and when it is still further proposed to introduce a whole series of exceptions into the obligation to notify which at present lies upon all medical men, we feel bound to protest against any such retrograde steps. The duty of notifying imposed upon practitioners is one of serious responsibility, and is often a very onerous and disagreeable task, for which he is ill rewarded by the trivial half-crown which is now so grudgingly paid to him. Many authorities, however, are greatly exercised by the fact that, when a second case of fever arises, the doctor gets another fee, and that, if the patients change their attendant during the progress of the illness, the second doctor also has to notify, and he also has to be paid, and such authorities are very anxious to dock off these duplicate notifications. It seems to be forgotten that the object of the Act is not merely to show where fever breaks out, but how far it prevails. At present the duty of notifying lies absolutely on every qualified practitioner, and it would be a great pity, and would certainly greatly detract from the utility and uniformity of the returns, if any exceptions were introduced. It is but a small and pettifogging saving which would be effected, and the result would be to make the returns entirely unreliable—to make them, in fact, depend upon the activity of the very authorities over whom, at the present time, they act as an independent check. If the sanitary authorities want to improve the working of the Act there is plenty of room for their intervention. So long as "dual notification" remains law, the law should be enforced. There can be no doubt that if the Legislature had not imposed the duty of notifying upon the householder, as well as upon the medical practitioner, some other means would have been invented for obtaining information in regard to those cases of infectious disease which are not under the care of qualified practitioners. As matters stand quacks and old women are outside the Act; and there is no doubt that these people are often asked to undertake the treatment of infectious diseases purely for the sake of avoiding notification, or that such disease is often spread broadcast from these hidden centres of infection. If we could see a few sanitary authorities taking the trouble to enforce that notification by householders which was made obligatory under the Act for the especial purpose of suppressing this very evil, we should have a little more faith than we have in their desire to make notification a reality.

